# Genome sequencing and metabolic network reconstruction of a novel sulfur-oxidizing bacterium *Acidithiobacillus Ameehan*

**DOI:** 10.3389/fmicb.2023.1277847

**Published:** 2023-11-20

**Authors:** Peng Wu, Qianqian Yuan, Tingting Cheng, Yifan Han, Wei Zhao, Xiaoping Liao, Lu Wang, Jingyi Cai, Qianqian He, Ying Guo, Xiaoxia Zhang, Fuping Lu, Jingjing Wang, Hongwu Ma, Zhiyong Huang

**Affiliations:** ^1^College of Bioengineering, Tianjin University of Science and Technology, Tianjin, China; ^2^Tianjin Key Laboratory for Industrial Biological Systems and Bioprocessing Engineering, Tianjin Institute of Industrial Biotechnology, Chinese Academy of Sciences, Tianjin, China; ^3^National Technology Innovation Center of Synthetic Biology, Tianjin, China; ^4^Biodesign Center, Key Laboratory of Systems Microbial Biotechnology, Tianjin Institute of Industrial Biotechnology, Chinese Academy of Sciences, Tianjin, China

**Keywords:** new sulfur-oxidizing bacteria, *Acidithiobacillus Ameehan*, genome-scale metabolic network model, pathways of cellular metabolism, constraint-based flux analysis

## Abstract

Sulfur-oxidizing bacteria play a crucial role in various processes, including mine bioleaching, biodesulfurization, and treatment of sulfur-containing wastewater. Nevertheless, the pathway involved in sulfur oxidation is highly intricate, making it complete comprehension a formidable and protracted undertaking. The mechanisms of sulfur oxidation within the *Acidithiobacillus* genus, along with the process of energy production, remain areas that necessitate further research and elucidation. In this study, a novel strain of sulfur-oxidizing bacterium, *Acidithiobacillus Ameehan*, was isolated. Several physiological characteristics of the strain Ameehan were verified and its complete genome sequence was presented in the study. Besides, the first genome-scale metabolic network model (AMEE_WP1377) was reconstructed for Acidithiobacillus Ameehan to gain a comprehensive understanding of the metabolic capacity of the strain.The characteristics of Acidithiobacillus Ameehan included morphological size and an optimal growth temperature range of 37-45°C, as well as an optimal growth pH range of pH 2.0-8.0. The microbe was found to be capable of growth when sulfur and K_2_O_6_S_4_ were supplied as the energy source and electron donor for CO_2_ fixation. Conversely, it could not utilize Na_2_S_2_O_3_, FeS_2_, and FeSO_4_·7H_2_O as the energy source or electron donor for CO_2_ fixation, nor could it grow using glucose or yeast extract as a carbon source. Genome annotation revealed that the strain Ameehan possessed a series of sulfur oxidizing genes that enabled it to oxidize elemental sulfur or various reduced inorganic sulfur compounds (RISCs). In addition, the bacterium also possessed carbon fixing genes involved in the incomplete Calvin-Benson-Bassham (CBB) cycle. However, the bacterium lacked the ability to oxidize iron and fix nitrogen. By implementing a constraint-based flux analysis to predict cellular growth in the presence of 71 carbon sources, 88.7% agreement with experimental Biolog data was observed. Five sulfur oxidation pathways were discovered through model simulations. The optimal sulfur oxidation pathway had the highest ATP production rate of 14.81 mmol/gDW/h, NADH/NADPH production rate of 5.76 mmol/gDW/h, consumed 1.575 mmol/gDW/h of CO_2_, and 1.5 mmol/gDW/h of sulfur. Our findings provide a comprehensive outlook on the most effective cellular metabolic pathways implicated in sulfur oxidation within *Acidithiobacillus Ameehan*. It suggests that the OMP (outer-membrane proteins) and SQR enzymes (sulfide: quinone oxidoreductase) have a significant impact on the energy production efficiency of sulfur oxidation, which could have potential biotechnological applications.

## Highlights

– Discovered a new sulfur-oxidizing bacterium *Acidithiobacillus Ameehan*.
–First experimentally verified genome-scale reconstruction metabolic network of *Acidithiobacillus Ameehan*.
– Clarify the metabolic mechanism of sulfur oxidation pathway coupled with carbon metabolism.

## 1. Introduction

Sulfur-oxidizing microorganisms (SOM) can oxidize elemental sulfur or various reduced inorganic sulfur compounds (RISCs) to sulfuric acid or higher valence sulfides. SOM not only play a vital role in the sulfur biogeochemical cycle, but are also widely used in the metallurgical industry, environmental engineering and agriculture (Chen et al., 2022). Several taxa of sulfur-oxidizing bacteria have been isolated and identified, including Green sulfur bacteria (GSB), Purple sulfur bacteria (PSB), Purple non-sulfur bacteria (PNSB), and Colorless sulfur bacteria (CSB; Yang and Shao, 2018). It is worth noting that *Acidithiobacillus*, one of the CSB, play a major role in bioleaching, biodesulfurization, sulfur-containing wastewater treatment, and so on (Wen et al., 2016; Singh et al., 2019). The genus *Acidithiobacillus* comprises eight recognized species, including *Acidithiobacillus ferrooxidans*, *Acidithiobacillus ferrivorans*, *Acidithiobacillus ferridurans*, *Acidithiobacillus ferriphilus*, *Acidithiobacillus ferrianus*, *Acidithiobacillus sulfuriphilus*, *Acidithiobacillus thiooxidans* (including *Acidithiobacillus albertensis*), and *Acidithiobacillus caldus* (also called *Fervidacidithiobacillus caldus*; Moya-Beltran et al., 2021). However, there are many species yet to be discovered (Poole, 2012; Moya-Beltran et al., 2021). All these *Acidithiobacillus* are aerobic, chemolithoautotrophic, gram-negative, non-sporulating, rod-shaped microorganisms (Kumar P. et al., 2019). They oxidize various RISCs to obtain electrons for their autotrophic growth. Several sulfur oxidation pathways have been speculated in *Acidithiobacillus* (Mangold et al., 2011; Talla et al., 2014; Wu et al., 2017; Moya-Beltran et al., 2021). In *Acidithiobacillus caldus*, extracellular elemental sulfur (S_8_) is transported into the periplasm by outer membrane proteins (OMP), where sulfur is oxidized to SO_3_^2−^. The SO_3_^2−^ can form S_2_O_3_^2−^ via a non-enzymatic reaction between SO_3_^2−^ and a sulfur atom. The SO_3_^2−^ and S_2_O_3_^2−^ can enter the sulfur oxidizing enzyme system (sox) pathway to form SO_4_^2−^ and elemental sulfur. The S_2_O_3_^2−^ can also be catalyzed by thiosulfate quinone oxidoreductase (TQO) to form S_4_O_6_^2−^ that is further hydrolyzed by tetrathionate hydrolase (TetH). The H_2_S generated in the transport of S_8_ is oxidized to elemental sulfur by sulfide quinone oxidoreductase (SQR) located in the inner membrane. The elemental sulfur is oxidized to SO_3_^2−^ by sulfur dioxygenase (SDO) or SOR-TST-HDR (SOR, sulfur oxygenase reductase; TST, rhodanese; HDR, HDR-like complex) located in the cytoplasm. The SO_3_^2−^ is further oxidized to SO_4_^2−^ via the APS pathway and SAT (ATP sulfurylase). *Acidithiobacillus ferrooxidans* does not have Sox system; its sulfur oxidation pathway is completely different from that of *Acidithiobacillus caldus. Acidithiobacillus ferrooxidans* equipped with Thiosulfate dehydrogenase (TSD), which could serve as an alternative Sox system. The quinol pool (QH2), located in the inner membrane, accepts electrons from TQO, HDR, and SQR. These electrons are then transferred to the terminal oxidases bd or bo3 for ATP production, or to NADH dehydrogenase (complex I) for NADH production (Wang et al., 2018). However, the sulfur oxidation pathway, particularly within the *Acidithiobacillus* genus, is a complex process that presents a significant challenge to fully comprehend. Despite some progress in characterizing certain genes and proteins, a comprehensive understanding of this pathway and its related metabolic processes remains elusive. Further research is needed to elucidate these mechanisms and the associated energy production (Hold et al., 2009; Koch and Dahl, 2018; Wang et al., 2018).

Genome-scale metabolic models (GEMs) are mathematical representations of metabolism for organisms and provide extensive gene–reaction–metabolite connectivity (Passi et al., 2022). GEMs combined with constraint-based algorithms, such as flux balance analysis (FBA; Orth et al., 2010), can be used to formulate mechanistic predictions of metabolic physiology (Orth et al., 2010; Larsen et al., 2012; Ang et al., 2018; Fang et al., 2020; Antoniewicz, 2021). There are over 6000 GEMs reconstructed for archaea, bacteria, and eukaryotes, which not only elucidate new biological knowledge and understanding (Gu et al., 2019; Chung et al., 2021; Oftadeh et al., 2021), but also help to design and engineer cellular metabolism (Kumar M. et al., 2019). Few GEMs have been used to analyze sulfur metabolism in *Acidithiobacillus*. The iMC507 model of *Acidithiobacillus ferrooxidans* ATCC 23270 is the only genome-scale metabolic network model (GEM) of *Acidithiobacillus* (Campodonico et al., 2016). TheiMC507 successfully elucidated the stoichiometry of proton translocation, electron transfer, and carbon flux distributions during chemolithoautotrophic growth of *Acidithiobacillus ferrooxidans* ATCC 23270 using Fe^2+^, S_4_O_6_^2−^, and S_2_O_3_^2−^. However, the iMC507 model only contain a fraction of the sulfur oxidation pathways of the *Acidithiobacillus* genus. Specifically, it includes the interconversion between S_4_O_6_^2−^ and S_2_O_3_^2−^, which generates an electron flow for cell growth and CO_2_ fixation. Moreover, the scope of this model is quite limited, comprising merely 615 reactions, 573 metabolites, and 461 genes. The pathway of element sulfur oxidation has not been thoroughly examined in iMC507 model. This is far from sufficient as a reference knowledge base for the extensive *Acidithiobacillus* genus. Furthermore, the primary focus of this model is on iron oxidation rather than sulfur oxidation. Therefore, there is an urgent need to develop a novel, more comprehensive model. Further investigation using GEM that involve additional sulfur oxidation reactions beyond what is represented in iMC507. This will improve our understanding of sulfur metabolism in *Acidithiobacillus*.

In this study, we isolated sulfur-oxidizing bacteria from a previous consortium 5Biol (Han et al., 2013). The isolated bacteria were subjected to various physical and chemical characterization tests, and then their entire genome was sequenced and analyzed. Furthermore, we employed the GEM to further investigate the sulfur oxidation pathway. This will provide a basis for future strain studies as well as aid in the investigation of the exact pathway of sulfur metabolism.

## 2. Materials and methods

### 2.1. Isolation of sulfur-oxidizing bacterium

Sulfur-oxidizing bacterium (SOB) was isolated from the pyrolusite leaching microbial community 5Biol (Han et al., 2013) using the 9 K-S solid medium at 37°C. The 9 K-S solid medium was composed of separately sterilized solution A, solution B, and 10.0 g/L sulfur powder (sterilized at 105°C for 24 h). To prepare solution A, 15.0 g agar was added to 250 mL 9 K medium (Silverman and Lundgren, 1959), and the pH was adjusted to 7.0 with H_2_SO_4_ (10 M) and NaOH (5 M). To prepare solution B, the pH of 750 mL 9 K medium lowered to 2.0.

### 2.2. Morphological characterization

Scanning electron microscopy (Hitachi SU8010, Japan) was used to perform morphological observation of the SOB. The SOB was grown aerobically in 9 K-S liquid medium with an initial pH of 2.0 at 37°C until the mid-exponential phase. The 9 K-S liquid medium was prepared by sterilizing 9 K medium and adding 10.0 g/L sulfur powder separately. To perform scanning electron microscopy (Hitachi SU8010, Japan), the samples were subjected to cell fixation, gradient dehydration, critical point drying (Leica EM CPD030, Germany), and gold coating (Hitachi E-1045, Japan) before being observed (Yang et al., 2023).

### 2.3. Conditions for optimal growth

The SOB with a 10% inoculation was cultured in 9 K-S liquid medium at various temperatures (15, 20, 25, 30, 37, 45, and 50°C) and with an initial pH of 2.0, maintaining a speed of 180 r/min. This experiment aimed to discover the optimal growth temperature of the strain. During the experiment, the OD_600_ was measured every 2 days to assess cell growth.

To investigate the optimal growth pH of SOB, the strain was grown at a constant temperature of 37°C and a rate of 180 r/min, using varied initial pH levels in the 9 K-S liquid medium (1.0, 2.0, 3.0, 4.0, 5.0, 6.0, 7.0, 8.0, and 9.0). As in the previous experiment, the OD_600_ was measured every 2 days to evaluate cell growth.

### 2.4. Sulfur oxidizing ability

To determine the sulfur-oxidizing ability of SOB, the strain was cultured in 9 K-S liquid medium with the optimal pH and temperature for 16 days. The samples were collected every 4 days. The SO_4_^2−^ was detected by a sulfate reagent powder pillow (HACH 2106769), and the pH was measured by a pH meter (pH meter FE20, China).

The SOB was cultured in 9 K medium containing a range of energy sources such as S (5.0 g/L), Na_2_S_2_O_3_ (10.0 g/L), K_2_S_4_O_6_ (1.5 g/L), FeS_2_ (10.0 g/L), FeSO_4_·7H_2_O (5.0 g/L), glucose (1.0 g/L), and yeast extract (0.2 g/L). The utilization of energy sources was assessed by monitoring the growth of the strain.

### 2.5. Growth experiments on various carbon sources

The ability of the strain to grow on different carbon sources was tested using the Biolog GEN III MicroPlate. According to the manufacturer’s instructions, a pure culture of the strain was incubated at 37°C, and then suspended in a special inoculating fluid at the predetermined cell density (90–98% transmittance). Then, 100 μL of the cell suspension was inoculated into each well of the GEN III MicroPlate™. The microplate was incubated at 37°C for 240 h, during which time kinetic information was recorded and quantified using Biolog’s GEN III OmniLog II ComboPlus kinetic software (Biolog, United States) followed by data analysis (Wragg et al., 2014). In the case of substrate utilization, photographic measurements of color intensity resulting from dye reduction were represented in OmniLog units (OU). The substrates with a Biolog value higher than 114 were considered positive.

### 2.6. Whole-genome sequencing

10 mL of the SOB in the mid-exponential phase was centrifuged at 10,000 rpm for 15 min. The resulting pellet was then washed twice with a sterile PBS solution containing 8.0 g/L NaCl, 0.2 g/L KCl, 1.44 g/L Na_2_HPO_4_, and 0.24 g/L KH_2_PO_4_. Genomic DNA was extracted from the pellet using the MOBIO PowerSoil® DNA Isolation kit. The quality and integrity of the extracted DNA were analyzed using NanoDrop2000 and 1% (w/v) agarose gel electrophoresis. The PacBio System based on single-molecule real-time (SMRT) sequencing technology (Wuhan Institute of biotechnology) was used for whole-genome sequencing. The sequencing data were assembled using SPAdes (v3.8.1; Bankevich et al., 2012). The whole genome sequence was deposited at NCBI GenBank under the accession number (CP118747–CP118752). All protein-coding genes were functionally annotated BLASTP (V2.2.3; Alex and Antunes, 2015) against the public protein sequence databases Kyoto Encyclopedia of Genes and Genomes (KEGG; Kanehisa et al., 2017) with an E-value ≤ 1e−5.

### 2.7. Phylogenomic analysis

To determine the ecological status of the strain, a phylogenetic tree was constructed using MEGA (version 11.0; Tamura et al., 2021) based on the 16S rRNA sequence. The evolutionary history was inferred using Maximum Likelihood (Tamura et al., 2004), based on the best-fitting model of nucleotide substitution using a General Time Reversible (GTR) model (Nguyen et al., 2015), and bootstrapped using 1,000 replicates. The inference was performed in MEGA, running for 1,000,000 generations and saving trees every 10,000 generations.

The average nucleotide identity (ANI; Arahal, 2014) was calculated using the FastANI tool (Jain et al., 2018). The results were used to generate the phylogenetic tree and heat map using the Seaborn and Matplotlib packages in Python (Hunter, 2007; Waskom, 2020). Euclidean was selected as the distance metric to use on the data. Selected “average” as the linking method to use to calculate clusters. Selected “1” for “standard_scaleint” to standardize the columns by subtracting the minimum and dividing each by its maximum.

### 2.8. Reconstruction of the genome-scale metabolic network

Four main steps were involved in constructing the GEM (Thiele and Palsson, 2010). To begin with, the genome of strain AMEEHan was annotated using the classicRAST and RASTtk pipelines available at https://rast.nmpdr.org (Overbeek et al., 2005; Brettin et al., 2015). The annotation results from two pipelines were automatically used for reconstructing models using ModelSEED available at https://modelseed.org (Overbeek et al., 2014). By integrating the model created by RASTtk annotations with the model created by classicRAST annotations, the draft model was reconstructed.

However, the draft model produced metabolites and ATP without providing any energetic substrate. The Parsimonious flux balance analysis (pFBA; Lewis et al., 2010) algorithm was utilized to identify pathways that resulted in a net production of ATP without supplying any carbon substrate, and the reversibility of reactions in draft model was corrected referring the MetaCyc database (Caspi et al., 2020). Afterward, the ATP production rate was examined when substrates were input.

The biomass composition of the gram-negative representative strain *E. coli* iML1515 (Monk et al., 2017) was adopted to represent the strain AMEEHan’s biomass. For simulating biomass synthesis, the same cell growth-associated and non-growth-associated ATP maintenance values (GAM and NGAM, respectively) as those used in iML1515 were assumed. After modifying the biomass composition, the biomass compositions that cannot be synthesized were identified. The optimal target for FBA calculation was set as the demand reaction of each biomass composition. A result of zero indicates that the biomass composition cannot be synthesized due to gaps in its synthesis pathway. The gaps for each composition were filled using the weight-added pFBA gap filling program (Luo et al., 2022).

Gap-filling was conducted for substrates that were applicable in the Biology experiment but were not available in the model. Two categories were identified: (1) Substrates that lack exchange reactions from extracellular to intracellular, which were filled manually. (2) Substrates that lack a utilization pathway, which were filled using the gap-filling program.

### 2.9. Validating model and simulating sulfur oxidation pathway

The uptake rate of the sulfur was set to 1.5 mmol/gDW/h, while the rates for other energy sources input were set to 0. The consistency and annotation of the model were evaluated using the metabolic model-testing suite (MEMOTE; Lieven et al., 2020). Flux balance analysis (FBA; Orth et al., 2010) was used to optimize a pre-defined objective function under specified metabolic constraints (Antoniewicz, 2021). If not otherwise specified, the objective function considered was the maximization of the biomass production rate. We selected parsimonious flux balance analysis (pFBA; Lewis et al., 2010) to analyze the biosynthesis pathway of the product. We conducted these methods using COBRApy (0.18.1; Ebrahim et al., 2013). The optimization solvers GLPK and Gurobi were used for linear and quadratic programming (Meindl and Templ, 2013).

An optimal sulfur oxidation pathway was predicted by maximizing the ATP production rate using FBA optimization. The model was iteratively computed by closing one of the reactions in the optimal sulfur oxidation pathway, which led to other possible differences in energy metabolic pathways.

## 3. Results

### 3.1. Physiological characterization of the SOB

**Figure 1:**
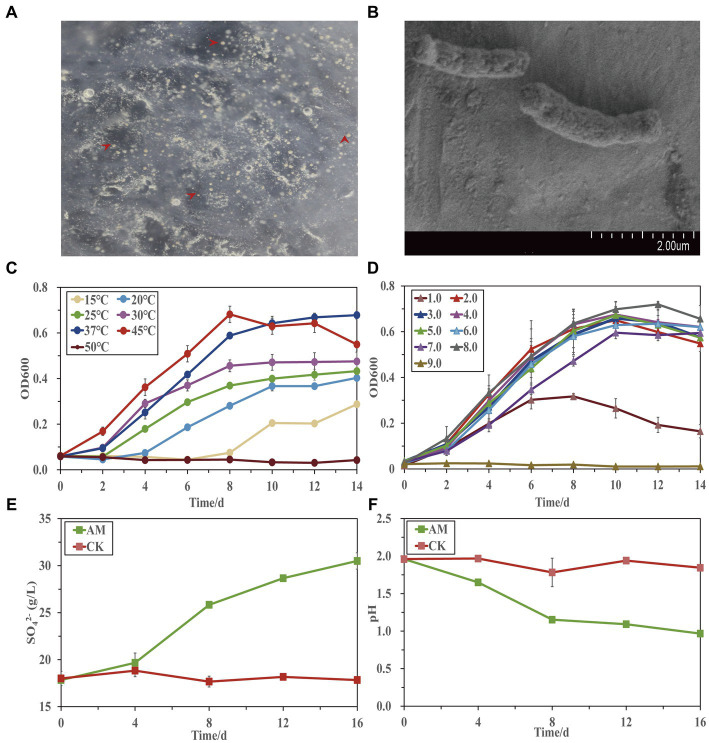
Physiological characterization of the strain AMEEHan. **(A)** The morphology of the strain. **(B)** Scanning electron microscopy (SEM) image of the strain. Effects of different temperatures **(C)** and pH **(D)** on the growth of the strain. Changes of SO_4_^2−^ concentration **(E)** and pH **(F)** during the strain growing in the 9 K-S liquid medium. CK, non-inoculation; AM, inoculated with the strain AMEEHan.

A sulfur-oxidizing bacterium, designated as strain AMEEHan, was successfully isolated from the pyrolusite leaching microbial community 5Biol (Han et al., 2013) using the 9 K-S solid medium at 37°C. The isolation was small, round, yellow, dome-shaped, and had a smooth surface with a distinct edge (Figure 1A). Scanning electron microscopy results demonstrated that the dimensions of the strain were approximately 1 μm × 2 μm and that it had a short rod-shaped (Figure 1B).

The strain exhibited growth within a temperature range of 15–45°C, with an increased growth rate as the temperature was sequentially raised from 15 to 45°C. The optimal growth temperatures for growth were found to be 37 and 45°C (Figure 1C). In terms of pH, the strain was able to grow within a range of pH 1.0–8.0. Growth was observed to be similar from pH 2.0 to 8.0, but was inhibited at pH 1.0 and pH 9.0 (Figure 1D).

When cultured under optimal conditions, the concentration of SO_4_^2−^ increased from 17 to 30 g/L while the pH decreased from 2.0 to 0.9 (Figures 1E,F). This indicated that approximately 50% sulfur was oxidized by the strain within 16 days.

The strain was found to be capable of growing on sulfur and K_2_O_6_S_4_, but not on Na_2_S_2_O_3_, FeS_2_, FeSO_4_·7H_2_O, glucose, and yeast extract (Table 1). Furthermore, it exhibited significantly enhanced growth on sulfur compared to K_2_O_6_S_4_, as indicated by higher OD600 values for sulfur compared to K_2_O_6_S_4_ (Supplementary Table S1).

**Table 1:** The energy utilization of strain AMEEHan.

Energy substances	OD_600_		
	0 days	3 days	6 days
Sulfur (5 g•L^−1^)	**0.002**	**0.163**	**1.645**
Na_2_S_2_O_3_ (10 g•L^−1^)	1.130	0.042	0.041
K_2_O_6_S_4_ (1.5 g•L^−1^)	**0.034**	**0.098**	**0.096**
FeS_2_ (10 g• L^−1^)	0.046	0.023	0.046
FeSO_4_ • 7 H_2_O (5 g•L^−1^)	0.046	0.041	0.041
Glucose (1 g•L^−1^)	0.045	0.067	0.069
Yeast extract (0.2 g•L^−1^)	0.045	0.046	0.037

Boid indicates that the strain AMEEHan can grow.

### 3.2. Genome analysis of strain AMEEHan

**Figure 2:**
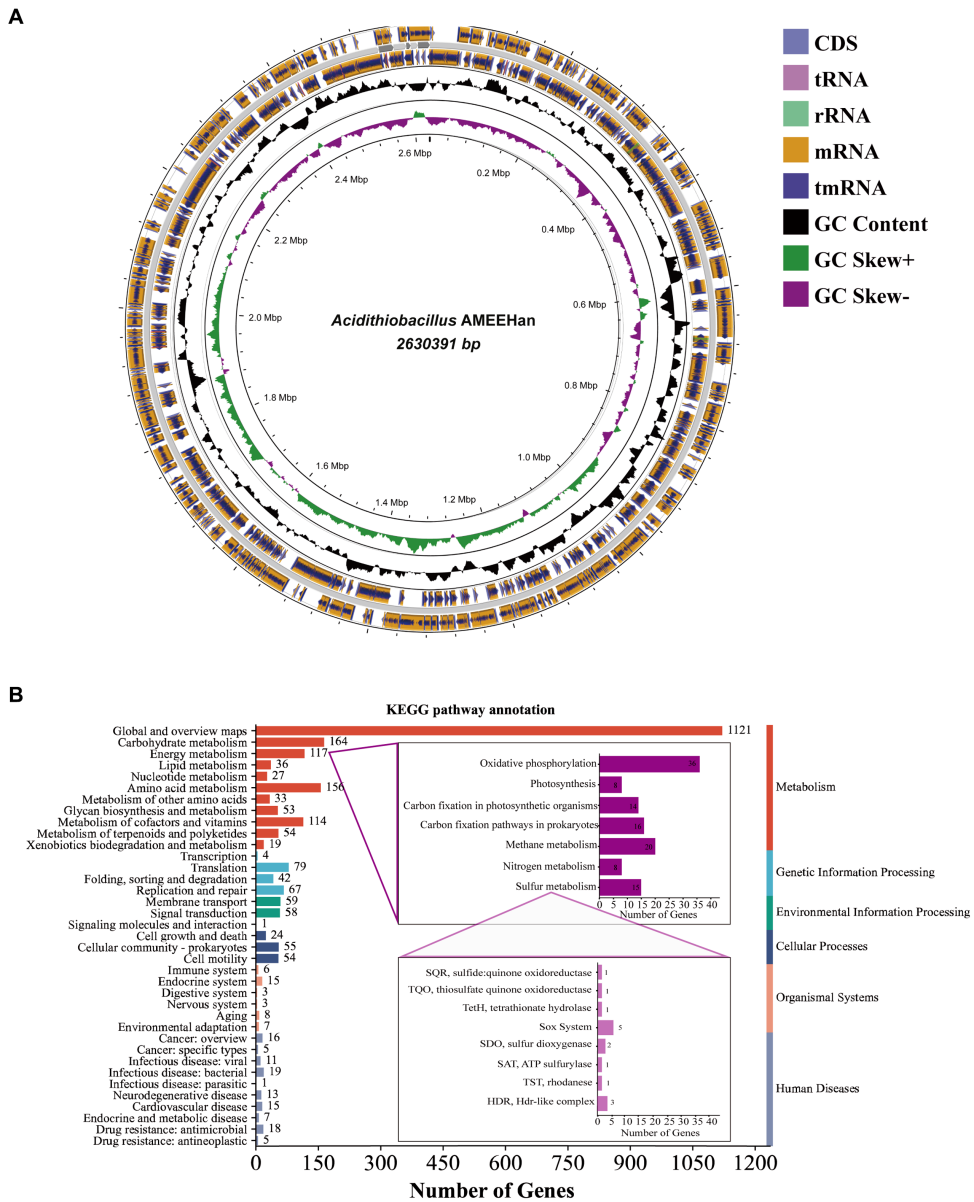
The genome of strain AMEEHan. **(A)** Circular genome map of the strain. The outermost circle represents the coding region on the positive and negative strands and the structural RNA genes, the innermost circle represents the variation in GC content in different regions of the genome, and the innermost circle represents the GC skew, where green indicates skew+ and purple indicates skew−. **(B)** A bar diagram of the number of genes involved in different functions from the KEGG pathway annotation.

The genome length of the strain consisted of a single circular chromosome of 2,630,391 bp with a G + C content of 58.6% (Table 2; Figure 2A). The genome encoded five plasmids, six ribosomal operons, 49 tRNA genes, and one tmRNA gene. A total of 2,582 protein-coding genes (CDSs) were predicted, of which 1,941 (75.2%) were assigned a putative function.

**Table 2:** Basic characteristics of the genome of strain AMEEHan.

Feature	Value
Genome length (bp)	2,630,391
GC%	58.6
Gene number	2,582
Genes with function prediction	1,941
Plasmid	5
rRNA	6
tRNA	49
tmRNA	1
16S rRNA	2

The majority of genes involved in global, carbohydrate, amino acid, energy, cofactors, and vitamins metabolism. In terms of energy metabolism, it primarily contained oxidative phosphorylation, methane metabolism, carbon fixation pathways in prokaryotes, and sulfur metabolism (Figure 2B). Fifteen genes were identified in sulfur metabolism, including SQR (sulfide: quinone oxidoreductase), thiosulfate quinone oxidoreductase (TQO), tetrathionate hydrolase (TetH), sox system, sulfur dioxygenase (SDO), SAT (ATP sulfurylase), TST (rhodanese), and HDR (HDR-like complex). These gene compositions suggested that the strain had the capacity to convert sulfur into sulfate. Additionally, it also exhibited the capacity for carbon fixation, as demonstrated by the presence of eight genes, which include fructose-bisphosphate aldolase (FBA), transketolase (tKt), ribose 5-phosphate isomerase A (rpiA), phosphoribulokinase (prk), Rubisco (ribulose-bisphosphate carboxylase small chain), phosphoglycerate kinase (pgk), glyceraldehyde 3-phosphate dehydrogenase (gap), and fructose-1,6-bisphosphatase (FBP; Supplementary Table S2).

**Figure 3:**
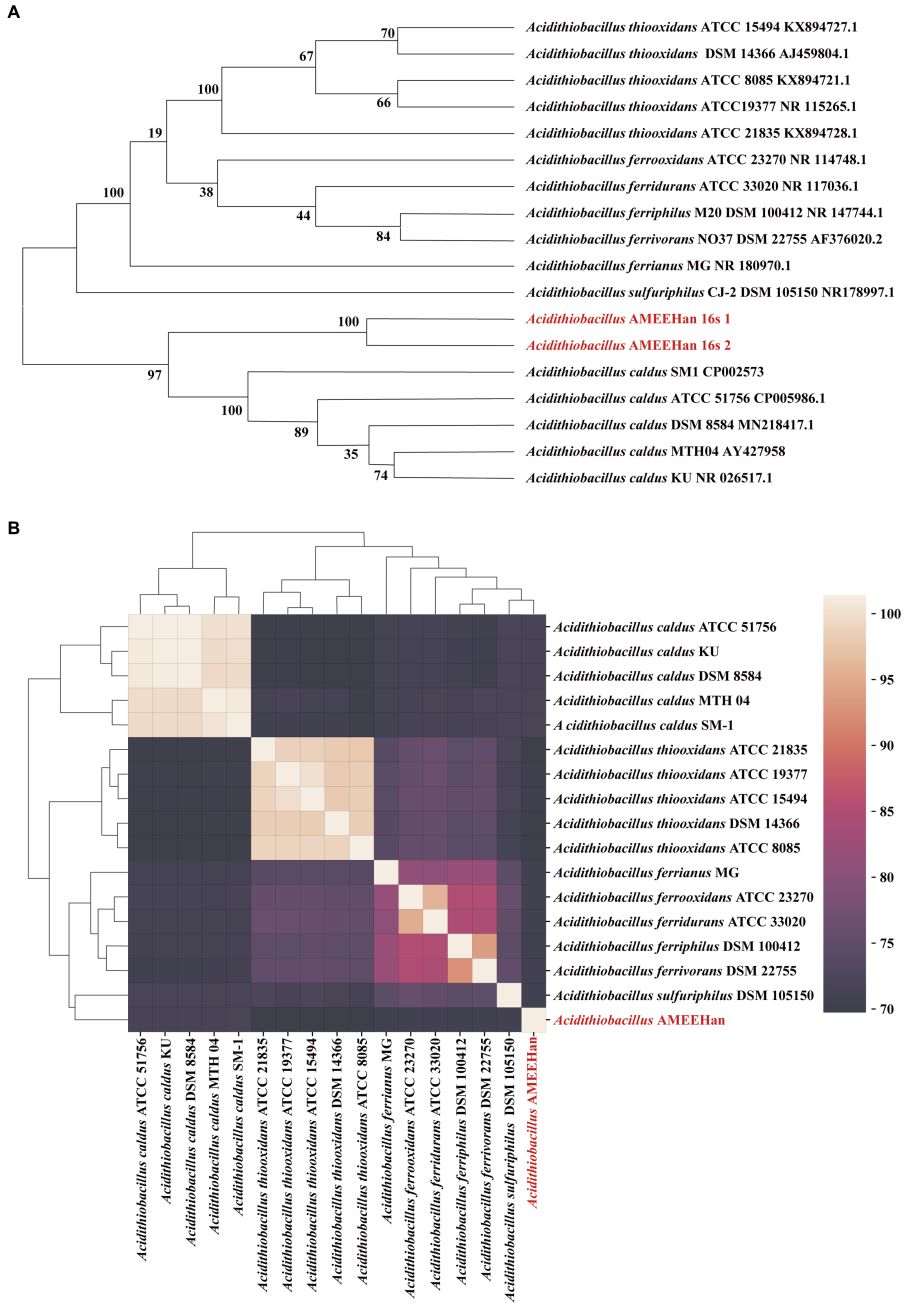
Classification of the strain AMEEHan. **(A)** Phylogenetic tree based on 16S rRNA gene by the Maximum Likelihood method. **(B)** Average Nucleotide Identity (ANI; %) based on the whole genomes.

Sixteen *Acidithiobacillus*, sharing high genetic similarity with strain AMEEHan, were utilized to create the 16S rRNA phylogenetic tree. The results illustrated that it had the highest degree of relatedness to *Acidithiobacillus caldus* SM1, with a similarity of 95.93% (Figure 3A). Furthermore, the same set of 16 *Acidithiobacillus* species that were used for the 16S rRNA analysis were evaluated for the ANI analysis. The ANI analysis indicated that the strain had the highest similarity to *Acidithiobacillus caldus* MTH 04 and *Acidithiobacillus caldus* SM-1, with ANI values of 70.38 and 70.29%, respectively (Figure 3B). The similarity of the strain to other strains was below 70% (Supplementary Table S3).

### 3.3. Reconstruction GEM of *Acidithiobacillus* AMEEHan

**Figure 4:**
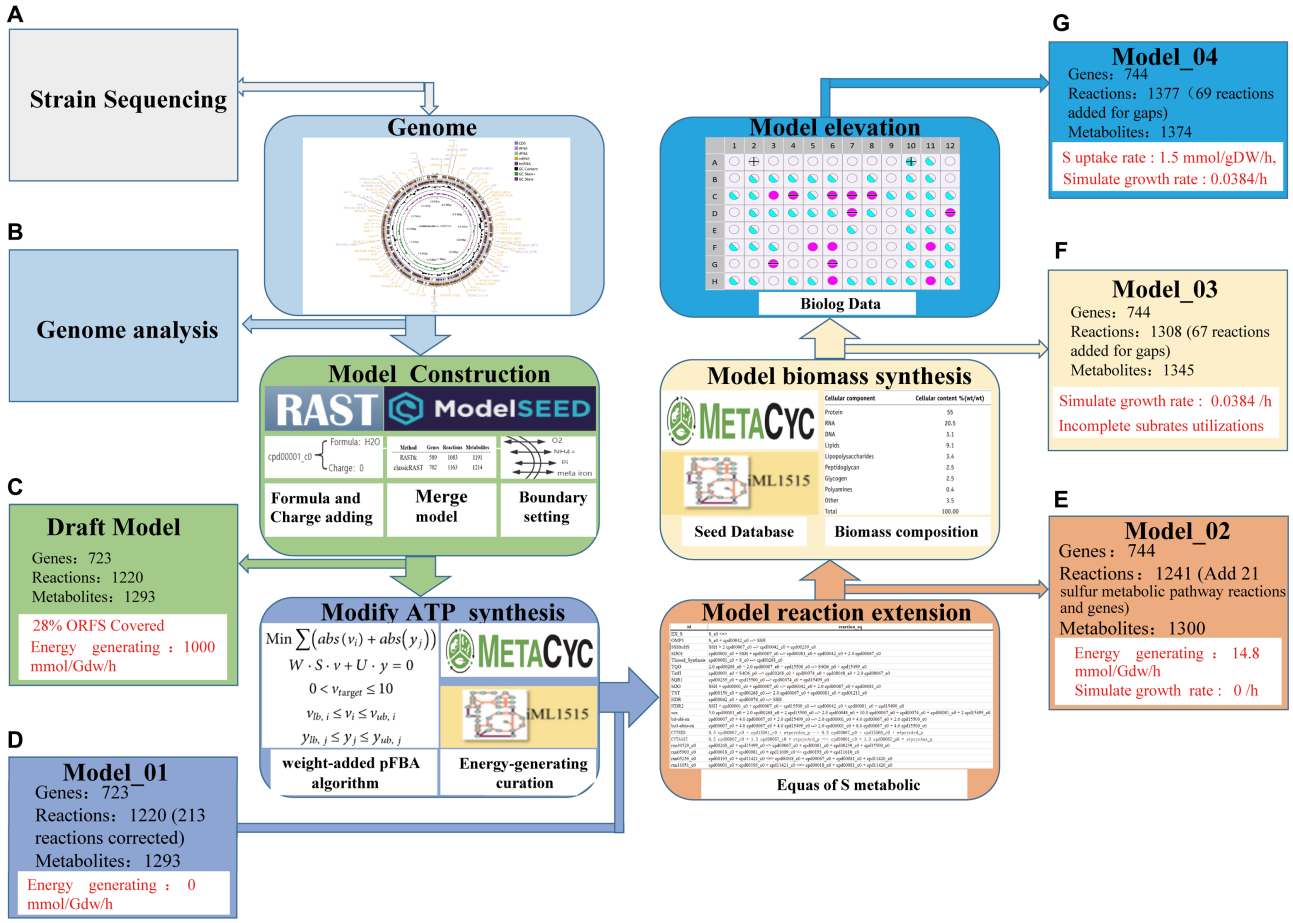
The reconstruction process of genome-scale metabolic network workflow for *Acidithiobacillus* AMEEHan. **(A)** Whole genome sequencing of the strain, perform whole genome sequencing of samples to obtain gene sequence data. **(B)** Analysis of bacterial whole genome, analyze genome sequences to obtain gene function annotation information. **(C)** Draft model generation and brief information, predict initial draft metabolic network model containing some gaps based on genome annotation. **(D)** Model_01 generation from Draft Model, refine and correct wrong reactions in Draft model using knowledge bases like Metacyc to produce Model_01. **(E)** Model_02 generation from Model_01, further integrate literature knowledge and add some known biochemical reaction pathways to generate Model_02. **(F)** Model_03 generation from Model_02, perform gap-filling on the Model_02 using the pFBA algorithm to produce Model_03. **(G)** Final model Model_04, based on Biolog experimental results, expand Model_03 to generate the final high-quality model Model_04.

The draft model of *Acidithiobacillus* AMEEHan was constructed by combining two models: one created with RASTtk annotation (589 genes, 1,191 metabolites, and 1,083 reactions), which covered 22% (589/2,708) of the annotated ORFs (Supplementary material; RASTtk, https://github.com/wupeng1998/Acidithiobacillus-Ameehan/tree/main/RASTtk) and the other with classicRAST annotation (702 genes, 1,214 metabolites, and 1,163 reactions), which covered 27% (702/2,582) of the annotated ORFs (Supplementary material; classicRAST, https://github.com/wupeng1998/Acidithiobacillus-Ameehan/tree/main/classicRAST). The information of charge and formula for the metabolites from ModelSEED were added to the draft model. The draft model comprised 723 genes, 1,293 metabolites, and 1,220 reactions, accounting for 28% (723/2,582) of the annotated ORFs (Figure 4C). Further analysis was conducted to assess the energy generation of the draft model. However, the simulated results showed the production of ATP without the supply of any carbon substrate (Figure 4C). To avoid a net production of ATP and correct the occurrence of energy-generating cycles (Supplementary Figure S1), 213 reactions were modified (Supplementary Table S4).

Following the above modifications, Model_01 was developed (Figure 4D). However, the ATP generation rate calculated by Model_01 was 0.0 mmol/gDW/h, when the sulfur uptake rate was 1.5 mmol/gDW/h. It indicated that there were gaps in the energy metabolism pathways. Through the integration of the physiological characteristics of the strain, genetic data, and an examination of the ATP synthesis pathway, it was ascertained that Model_01 exhibited a malfunctioning sulfur oxidation pathway. Therefore, the Model_01 was extended to generate Model_02 by incorporating 21 additional reactions and seven new metabolites (Supplementary Table S5) based on the genome of the strain and other reports (Wang et al., 2018). The optimum ATP generation rate calculated by Model_02 was 14.8 mmol/gDW/h with the sulfur uptake rate of 1.5 mmol/gDW/h (Figure 4E).

The growth rate simulated by Model_02 was zero with the same sulfur uptake rate. However, the experimentally measured growth rate of *Acidithiobacillus* AMEEHan was 0.0379 h^−1^ when cultured in minimal medium (Supplementary Table S4). Upon analyzing the biomass composition, it was revealed that the inability to synthesize certain biomass precursors was the reason why Model_02 failed to simulate growth accurately. In order to tackle this problem, the synthetic pathway for nine precursors was completed by adding a total of 68 reactions (Supplementary Table S6), which resulted in the development of Model_03 (Figure 4F). Model_03 predicted a growth rate of 0.0384 h^−1^, which was close to the actual *in vivo* value of 0.0379 h^−1^ (Supplementary Figure S2).

**Figure 5:**
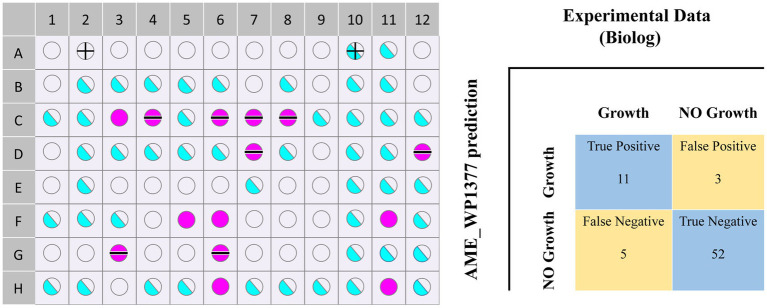
The Biolog carbon source validation results in models and experiments. Purple circles indicate a positive value, while blue circles indicate a boundary value. Blank circles indicate negative values, and semi-blue circles indicate boundary values. Wired circles suggest that the comparison results with *Corynebacterium pilosum* in the Biolog GEN III MicroPlate™ library are false positive or false negative. However, as there are no Acidithiobacillus strains present in the Biolog GEN III MicroPlate™ library, false negative values are considered negative and false positive values are considered positive.

The Biolog phenotype microarray experiments were conducted, revealing that 16 of 71 carbon sources can be used by *Acidithiobacillus* AMEEHan (Supplementary Table S7). However, 15 of the 16 carbon sources could not be utilized by Model_03 due to the lack of transport reactions or utilization pathways. For instance, D-Mannose had an exchange reaction in model_03, but its metabolic pathway was missing one step, “D-mannose-6-phosphate = > D-fructose-6-phosphate.” Model_04 was generated after supplementing 70 reactions (Figure 4G; Supplementary Table S8). Model_04 demonstrated an 87.5% agreement with the experimental data, yielding 11 true positive (substrates that can be used both *in vivo* experiments and simulations) and 52 true negative (substrates that cannot be used both *in vivo* experiments and simulations) results (Figure 5; Supplementary Table S7).

**Figure 6:**
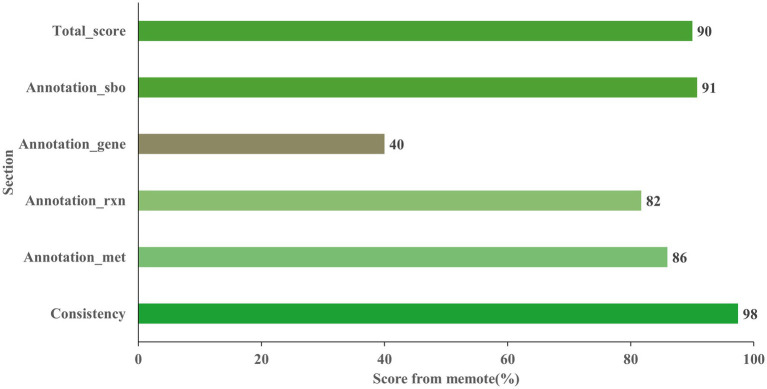
Evaluation the *Acidithiobacillus* AMEEHan model AME_WP1377 by MEMOTE.

The final model Model_04, named AME_WP1377 (Model_04), was comprised of 744 genes, 1,374 metabolites, and 1,377 reactions that are distributed among the extracellular, periplasmic, and cytoplasmic compartments^1^. The model of AME_WP1377 was evaluated by MEMOTE (Lieven et al., 2020; Supplementary material—MemoteReportApp.html). The annotation process evaluated the assignment of appropriate SBO terms to model instances using the Systems Biology Ontology (SBO) terminology. The SBO annotation achieved a high score of 91%. The annotations for reactions and metabolites achieved scores of 82 and 86%, respectively. The model consistency check consisted of tests to evaluate stoichiometric consistency, mass and charge balance, metabolite connectivity, and unbounded fluxes in the default medium. Moreover, the consistency score of AME_WP1377 was high at 98%. These results indicate a clear advantage of AME_WP1377 with a total score of 90% (Figure 6).

### 3.4. Simulating optimal sulfur oxidation pathway of *Acidithiobacillus* AMEEHan

Through model simulation, the AME_WP1377 model demonstrated the existence of five potential pathways for sulfur oxidation in *Acidithiobacillus* AMEEHan when using sulfur as substrates (Table 3). By consuming 1.5 mmol/gDW/h sulfur, it in the path I can produce 14.81 mmol/gDW/h ATP, 5.76 mmol/gDW/h NADH/NADPH, and fixation CO_2_ at a rate of 1.575 mmol/gDW/h with the growth rate of 0.0384 mmol/gDW/h. In path II, it can produce 6.56 mmol/gDW/h ATP, 2.76 mmol/gDW/h NADH/NADPH and fix CO_2_ at a rate of 0.694 mmol/gDW/h with the growth rate of 0.0169 mmol/gDW/h. In path III, it can produce 5.63 mmol/gDW/h ATP, 2.13 mmol/gDW/h NADH/NADPH, and fix CO_2_ at a rate of 0.598 mmol/gDW/h with the growth rate of 0.0146 mmol/gDW/h. In path IV, it can produce 4.69 mmol/gDW/h ATP and 1.50 mmol/gDW/h NADH/NADPH and fix CO_2_ at a rate of 0.499 mmol/gDW/h with the growth rate of 0.0121 mmol/gDW/h. In path V, it can produce 2.25 mmol/gDW/h ATP and 0.95 mmol/gDW/h NADH/NADPH and fix CO_2_ at a rate of 0.238 mmol/gDW/h with the growth rate of 0.0058 mmol/gDW/h.

**Table 3:** The calculated five sulfur oxidation pathways of *Acidithiobacillus* AMEEHan.

Sulfur oxidation pathways	NADH/NADPH production rate (mmol/gDW/h)	ATP production rate (mmol/gDW/h)	CO_2_ uptake rate (mmol/gDW/h)	Growth rate (mmol/gDW/h)
Path I	5.76	14.81	1.575	0.0384
Path II	2.76	6.56	0.694	0.0169
Path III	2.13	5.63	0.598	0.0146
Path IV	1.50	4.69	0.499	0.0121
Path V	0.95	2.25	0.238	0.0058

**Figure 7:**
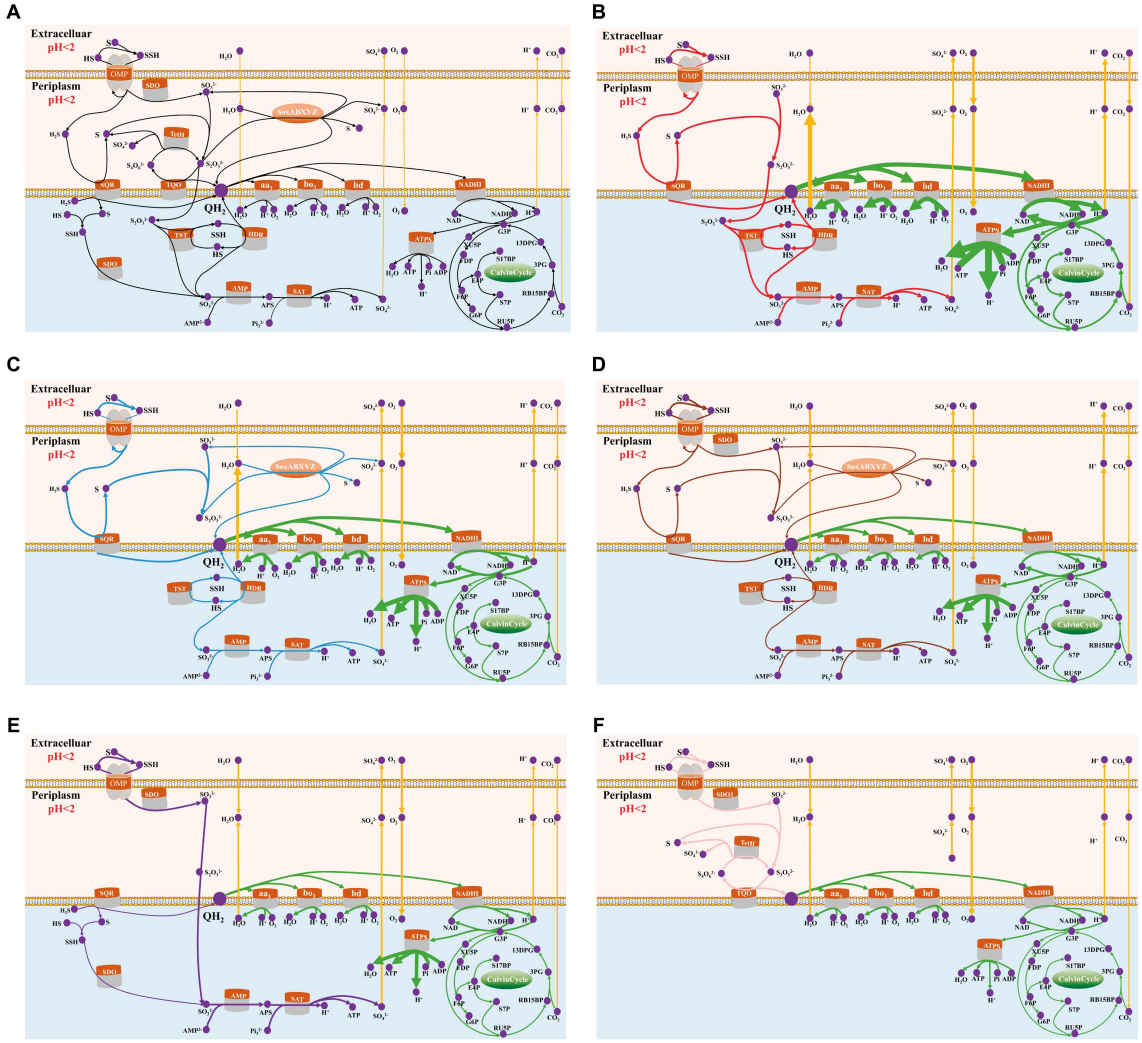
Sulfur oxidation pathway of *Acidithiobacillus* AMEEHan *in silico* predictions. The green line showed the common sulfur oxidation reactions found in all pathways while the yellow lines indicated intracellular and extracellular transport of substances. The purple dots represented the corresponding metabolites. Arrows indicate the direction of enzymatic activity and the arrow thicknesses are proportional to the flux through each reaction (a thicker arrow has a larger flux). **(A)** The potential sulfur oxidation pathways in *Acidithiobacillus* AMEEHan. **(B)** The red line denoted the optimal pathway predicted for sulfur oxidation. **(C)** The blue line denoted the second pathway predicted for sulfur oxidation. **(D)** The brown line denoted the third pathway predicted for sulfur oxidation. **(E)** The purple line denoted the fourth pathway predicted for sulfur oxidation. **(F)** The pink line denoted the fifth pathway predicted for sulfur oxidation. S, Sulfur; SSH, Sulfane sulfur atom of glutathione persulfide; OMP, Outer-membrane proteins; TQO, Thiosulfate quinone oxidoreductase; TetH, Tetrathionate hydrolase; SoxABXYZ, Sulfur oxidizing enzyme system; SQR, Sulfide: quinone oxidoreductase; SDO, Sulfur dioxygenase; TST, Rhodanese; HDR, HDR-like complex; SAT, ATP sulfurylase; aa3, bd, bo3, terminal oxidases; QH2, Quinol pool; NADHI, NADH dehydrogenase complex I; and ATPs, ATP hydrolytic enzyme.

By model simulation, from the potential sulfur oxidation pathways in *Acidithiobacillus* AMEEHan (Figure 7A), an optimal sulfur oxidation pathway (Table 3, Path I; Figure 7B) had been predicted. In Path I, the extracellular elemental sulfur (S) was activated to sulfide and transported by special outer-membrane proteins (OMP) with sulfide into the periplasm. In the periplasm, the sulfide was oxidized to produce H_2_S, which was further oxidized by the SQR enzyme (sulfide: quinone oxidoreductase), located in the inner membrane, to generate sulfur and QH_2_. The sulfur reacted with SO_3_^2−^ in a conventional chemical reaction to S_2_O_3_^2−^. Subsequently, the TST enzyme (rhodanese enzyme) and HDR enzymes (Hdr-like complex enzymes) catalyze the S_2_O_3_^2−^ to produce SO_3_^2−^ and QH_2_. Finally, SO_3_^2−^ is processed through the APS pathway to generate ATP and SO_4_^2−^, and the SO_4_^2−^ was secreted out of the cell. Additionally, the electron produced by sulfur oxidation were stored in QH_2_, which reduced O_2_ through the downhill pathway of the ubiquinone cytochrome oxidoreductase complex (cytochrome bd, bo_3_, and aa_3_ complex), and reduces NAD/NADP through the uphill pathway of NADHI, resulting in the production of reducing power NADH. The produced NADH participated in the incomplete Calvin–Benson–Bassham (CBB) cycle to fix environmental CO_2_ (Figure 7B).

The second alternative sulfur oxidation pathway (Table 3, Path II) is the same as in the first pathway from S to S_2_O_3_^2−^, followed by oxidation of S_2_O_3_^2−^ by the Sox system (SoxABXYZ) in the periplasmic space to monomeric sulfur in the cytoplasmic for sulfur oxidation in the first pathway. As for electron transfer to generate proton pumps, NADH regeneration and CO_2_ fixation are consistent with the first sulfur oxidation pathway (Figure 7C).

A third potential pathway (Table 3, Path III) for sulfur oxidation is almost similar to the second pathway, except that extracellular elemental sulfur enters the cell and is simultaneously oxidized by SQR and SDO into the periplasmic space for sulfur oxidation (Figure 7D).

The fourth alternative sulfur oxidation pathway (Table 3, Path IV) is activation of extracellular elemental sulfur through OMP and transport to the periplasm, where it is converted to SO_3_^2−^ by SDO enzyme. The resulting SO_3_^2−^ is transported from the periplasm to the cytoplasm by a transport system. As for electron transport, H_2_S in the cytoplasm is oxidized by the SQR, located in the inner membrane, to produce sulfur and QH_2_. The SSH is oxidized by SDO enzyme in the cytoplasm to produce SO_3_^2−^. Finally, the SO_3_^2−^ is processed through the APS pathway to generate ATP and SO_4_^2−^, and the SO_4_^2−^ is secreted out of the cell through the transport system (Figure 7E).

The fifth alternative sulfur oxidation pathway (Table 3, Path V) involves activation of extracellular elemental sulfur through OMP and transport to the periplasm, where it is oxidized by the SDO enzyme to SO_3_^2−^. The sulfur reacts with SO_3_^2−^ in a conventional chemical reaction to S_2_O_3_^2−^. This compound is further oxidized by TQO to S_4_O_6_^2−^ and QH_2_. The S_4_O_6_^2−^ is converted by TetH to S_2_O_3_^2−^, sulfur and SO_4_^2−^. Finally, the SO_4_^2−^ is secreted out of the cell through the transport system (Figure 7F).

## 4. Discussion

### 4.1. The *Acidithiobacillus* AMEEHan is a new species

The physicochemical properties of strain AMEEHan were significantly different from other species of the genus *Acidithiobacillus* (Table 4). The growth pH of the strain ranged from 2.0 to 8.0, while other species of the genus *Acidithiobacillus* ranged from 1.7 to 4.0 (Kelly and Wood, 2000; Kupka et al., 2007; Valdes et al., 2011; Wang et al., 2018; Sriaporn et al., 2021). This broader optimal pH range suggested that strain AMEEHan possessed a heightened ability to adapt to varying pH conditions, thereby enhancing its competitive advantage across different growth pH levels, which would provide great application potential for its future application. More importantly, all species of the genus *Acidithiobacillus* but not *Acidithiobacillus* AMEEHan were able to utilize S_2_O_3_^2−^ as energy source (Bryant et al., 1983; Johnson, 1998; Janiczek et al., 2007; Valdes et al., 2008; Beard et al., 2011; Chen et al., 2012; Hedrich and Johnson, 2013a,b; Yin et al., 2014; Falagán and Johnson, 2016; Miyauchi et al., 2018; Zhan et al., 2019; Zhang et al., 2020; Moya-Beltran et al., 2021; Zhao et al., 2021). Five plasmids were detected in the strain, while there were less than four plasmids in other *Acidithiobacillus* (*Acidithiobacillus ferrooxidans* YQ-N3 contained five plasmids; Valdes et al., 2009; You et al., 2011; Moya-Beltran et al., 2021). These indicated that the strain might be a new species of the genus *Acidithiobacillus.*

**Table 4:** Growth phenotypic features of species in the genus of *Acidithiobacillus.*

	*A. ferrooxidans* (Janiczek et al., 2007; Beard et al., 2011; Hedrich and Johnson, 2013b; Zhan et al., 2019; Zhang et al., 2020)	*A. ferrivorans* (Hedrich and Johnson, 2013a,b; Zhao et al., 2021)	A. *ferridurans* (Hedrich and Johnson, 2013a; Miyauchi et al., 2018)	A. *ferriphilus* (Falagán and Johnson, 2016)	A. *ferrianus* (Norris et al., 2020)	A. *sulfuriphilus* (Falagan et al., 2019)	*A. thiooxidans* (Bryant et al., 1983; Johnson, 1998; Valdes et al., 2011; Yin et al., 2014; Falagán and Johnson, 2016; Moya-Beltran et al., 2021)	*A. caldus* (Valdes et al., 2009; Tapia et al., 2012; Moya-Beltran et al., 2021)	A. *Ameehan*
Gram stain	−	−	−	−	−	−	−	−	−
Cell size (μm)	1.0 × 0.5	2.4 × 0.5	1.0–2.0	1.0–2.0	1.2–2.5	1.5–2.5	1.0–2.0	1.2–1.9	1.0–2.0
Motility	−	+	+	+	+	+	+	+	+
Growth pH (Optimum)	1.3–4.5 (2.0–2.5)	1.9–3.4 (2.5)	1.4–3.0 (2.1)	1.5 (2.0)	1.7–3.5 (1.7–2.0)	1.8–7.0 (3.0)	0.5–5.5 (2.0–3.0)	0.5–6.0 (2.0–4.0)	1.0–8.0 (2.0–8.0)
Growth T (°C) (Optimum)	10–37 (30–35)	4–37 (28–33)	10–37 (29)	5–33 (30)	28–32 (20–32)	4–45 (25–28)	18–37 (28–30)	10–52 (25–45)	15–45 (37–45)
Oxidation of sulfur	+	+	+	+	+	+	+	+	+
Oxidation of S_4_O_6_^2−^	+	+	+	+	+	+	+	+	+
Oxidation of S_2_O_3_^2−^	+	+	+	+	+	+	+	+	−
Oxidation of Fe^2+^	+	+	+	+	+	−	−	−	−
Growth on sulfide minerals	+	+	+	+	+	NR	−	−	−
Anaerobic growth with Fe^3+^	+	+	+	+	+	−	−	−	−
N_2_ fixation	+	+	NR	NR	NR	NR	−	−	−
Plasmids	No more five	No more Two	No plasmid	No more Two	No plasmid	No plasmid	No plasmid	No more Two	Five plasmids
G + C content (%)	58–59	55–56	58.4	57.4	56–59	61.5	52	61.5–64	58.6

A, Acidithiobacillus; NR, Not report; +, positive; −, negative.

The 16S rRNA gene phylogenetic tree showed that *Acidithiobacillus* AMEEHan was closest to *Acidithiobacillus caldus* SM-1 with 95.68% (Figure 3A). However, the 16S rRNA genes with more than 97% or even 99% similarity represent the same species (Gevers et al., 2005; Edgar, 2018).

Although the 16S rRNA gene has been a useful marker for species classification, this conserved gene approach has limitations in large-scale taxonomy (Huse et al., 2008; Klindworth et al., 2013; Jeong et al., 2021). To address these limitations, we use the ANI value to compare genomes in pairs, which has higher resolution and stronger correlation (Richter and Rossello-Mora, 2009; Vandervalk et al., 2015). The ANI comparisons showed that the maximum similarity between the strain and the genus *Acidithiobacillus* were no more than 70%. However, the ANI values of the same species should be higher than 95% (Arahal, 2014; Yoon et al., 2017). The strain AMEEHan is a novel species in the genus *Acidithiobacillus*, as demonstrated by these results, which highlights its significant divergence from other strains in the genus. We have therefore named it *Acidithiobacillus Ameehan*.

### 4.2. *Acidithiobacillus Ameehan* had unique functional genes and pathways

**Figure 8:**
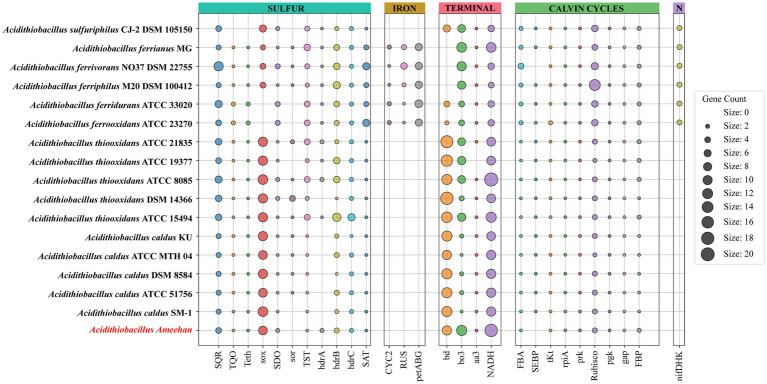
Metabolic characteristics of *Acidithiobacillus Ameehan* and other species of the *Acidithiobacillus*. The genes annotated for each strain of the genus *Acidithiobacillus* in sulfur metabolism, carbohydrate metabolism, metal iron oxidation and nitrogen fixation.

Similar to *Acidithiobacillus thioooxidans* and *Acidithiobacillus caldus*, *Acidithiobacillus Ameehan* lacks the ability to oxidize iron and fix nitrogen (Table 4). A putative nitrogenase gene cluster (nifDHK) of *Acidithiobacillus Ameehan* was found similar to that of most other *Acidithiobacillus* except *Acidithiobacillus caldus* and *Acidithiobacillus thiooxidans* (Figure 8). These genes may encode the nitrogenase complex and proteins involved in the synthesis of the nitrogenase cofactor.

The genomes of *Acidithiobacillus* species contained genes encoding enzymes and electron transfer proteins predicted to be involved in the oxidation of RISCs (Figure 8; Supplementary Table S2). Notably, the functional genes of *Acidithiobacillus Ameehan* were significantly different from other *Acidithiobacillus* species. *Acidithiobacillus Ameehan*, similar to *Acidithiobacillus caldus* SM-1 and *Acidithiobacillus thiooxidans* ATCC 19377, possessed 10 out of 11 genes related to sulfur metabolism, with the exception of the *sor* gene. The *sor* gene was a key enzyme in archaea that was often used for energy production from inorganic sulfur oxidation, but was found to be nonessential in *Acidithiobacillus* (Chen et al., 2012; Ferreira et al., 2022). The *sor* gene deletion was also simulated in the AME_WP1377 model. It had no effect on the growth of the strain when the *sor* gene (4 H_2_O + O_2_ + 4 Elemental-Sulfur = > 4 H^+^ + 2 Sulfite +2 H_2_S) was added to the model (Supplementary Table S9). Therefore, the loss of this unnecessary gene in *Acidithiobacillus Ameehan* is likely a result of long-term natural evolution and adaptation to its specialized niche.

The genus *Acidithiobacillus* fixed CO_2_ via the CBB reductive pentose phosphate cycle, using energy and reducing power derived from the oxidation of iron or sulfur. However, it is worth noting that both *Acidithiobacillus Ameehan* and *Acidithiobacillus ferrivorans* NO37 DSM 22755 were found to lack the enzyme SEBP (sedoheptulose-1,7-bisphosphatase) in the CBB. The enzyme should catalyze the reaction of sedoheptulose-1,7-bisphosphatase to sedoheptulose-7-phosphate during the regeneration of RuBP in the third steps of the CBB cycle (Supplementary Figure S3). This absence suggests that these organisms may have alternative mechanisms to compensate for the lack of SEBP function. Interestingly, it was observed that the enzyme 2.2.1.2, which converts erythrose-4-phosphate to sedoheptulose-7-phosphate, known as dihydroxyacetone transferase, could potentially fulfill the role of SEBP, and provide a viable substitute in the metabolic pathway (Figure 8). Model simulations can provide a solid explanation for this discrepancy. In model simulations, we discovered that when *Acidithiobacillus Ameehan* used the incomplete CBB cycle, they were able to fix more CO_2_, and the growth rate was better than the classical CBB cycle (Table 5). This discovery may provide some theoretical insight into the evolution of the strain.

**Table 5:** Differences between classical CBB and incomplete CBB cycles.

	S uptake rate (mmol/gDW/h)	CO_2_ fixation rate (mmol/gDW/h)	Growth rate (mmol/gDW/h)
Classical CBB	1.5	1.455	0.0355
*Acidithiobacillus Ameehan* CBB	1.5	1.575	0.0384

And for K_2_S_4_O_6_ utilization, when simulated with K_2_S_4_O_6_ as the energy substrate at the same CO_2_ uptake rate (1.575 mmol/gDW/h), the model showed that the K_2_S_4_O_6_ uptake rate was 0.76 mmol/gDW/h. This was equivalent to elemental sulfur at a rate of 3.04 mmol/gDW/h. It indicated that *Acidithiobacillus Ameehan* had a relatively low efficiency in the utilization of K_2_S_4_O_6_, which was consistent with the experimental results (Table 1). Comparing the energy consumed by the AMEE_WP1377 model and the *Acidithiobacillus ferrooxidans* ATCC 23270 model iMC507 (Campodonico et al., 2016) under the same fixed CO_2_ rate, we found that the oxidation efficiency of *Acidithiobacillus Ameehan* for RISCs was higher than that of *Acidithiobacillus ferrooxidans* ATCC 23270. The oxidation efficiency of *Acidithiobacillus Ameehan* on S_4_O_6_^2−^ (S_4_O_6_^2−^ uptake rate was 0.717 mmol/gDW/h) was about six times higher than that of *Acidithiobacillus ferrooxidans* ATCC 23270 (S_4_O_6_^2−^ uptake rate was 4.45 mmol/gDW/h; Eccleston and Kelly, 1978; Campodonico et al., 2016). These results further demonstrated the high application value of *Acidithiobacillus Ameehan* in bioleaching.

### 4.3. Simulation of the optimal sulfur oxidation pathway of *Acidithiobacillus Ameehan*

The sulfur oxidation pathway is extremely complex, and unraveling it will be a long and difficult endeavor. Although some genes and proteins involved in sulfur oxidation have been characterized, there is still a lack of accurate and comprehensive analysis of the sulfur oxidation pathway and its related metabolic pathways (Wang et al., 2018; Yang et al., 2019). However, by using GEMs, we can obtain an optimal sulfur oxidation pathway (Figure 7B), which provides a clearer understanding of the sulfur oxidation pathway in the study of *Acidithiobacillus Ameehan*. By examining the changes in the oxidation states of elemental sulfur during the energy production process in these five calculated sulfur metabolic pathways, we discovered that the first pathway produced the highest amount of energy. This could be due to its ability to completely oxidize all elemental sulfur to the final sulfate state. However, the other proposed pathways did not completely oxidize all elemental sulfur to the final sulfate state. In addition, the OMP protein played a critical role in all sulfur oxidation pathways because all sulfur oxidation pathways required it to transport extracellular elemental sulfur into the cell. Meanwhile, enzymes in other pathways could discover possible alternative pathways or alternative enzymes, increasing the resilience of the pathways. In addition, we found that SQR enzymes were involved in the top three pathways with the highest energy production efficiency. This suggests that SQR enzymes have a significant impact on the energy production efficiency of sulfur oxidation. It uses inorganic electron donor (sulfur) to generate reducing power to drive light-dependent CO_2_ fixation via the incomplete CBB cycle. This discovery could serve as a valuable breakthrough and reference point for future strain modifications aimed at improving sulfur oxidation efficiency.

While the above results are based on computational modeling, further experimental validation will be critical in the future. The genome-scale metabolic modeling approach allowed for the identification of key enzymes and the optimal sulfur oxidation pathway, providing valuable hypotheses to explore experimentally. Future investigations should prioritize validation of the predicted sulfur oxidation pathway and quantification of flux through said pathway. The functions of crucial enzymes, including SQR, need to be verified through experiments, such as enzyme assays and gene knockouts. Moreover, the comparative energetics of various putative sulfur oxidation pathways could be assessed by measuring growth and ATP production in mutants that lack different enzymes. Following these experimental approaches, apart from confirming computational results, can also provide additional insights into sulfur oxidation mechanisms in *Acidithiobacillus Ameehan*. Combining genome-scale modeling with targeted experiments will enhance our understanding of this intricate and ecologically significant metabolic process.

## 5. Conclusion

In this research investigation, a sulfur-oxidizing bacterium was isolated from the 5biol colony. The complete genome sequence of this bacterium was determined and subsequently used to confirm various physiological traits of the isolation. These characteristics included a small, round, yellow, dome-shaped, and had a smooth surface with obvious edges. The strain had a size of 1 μm × 2 μm, and a short rod-shaped. Moreover, it demonstrated an optimal growth temperature range of 37–45°C and an optimal growth pH range of pH 2.0–8.0. The microbe was able to grow on sulfur and K_2_O_6_S_4_, but could not grow on Na_2_S_2_O_3_, FeS_2_, FeSO_4_·7H_2_O, glucose, or yeast extract. Phylogenetic tree construction based on the 16S rRNA and whole-genome average nucleotide identity (ANI) values of the strain allowed us to identify the isolated strain as a novel strain, named *Acidithiobacillus Ameehan*. Furthermore, the KEGG pathway annotated several genes associated with distinct functions. This strain was capable of oxidizing elemental sulfur or different RISCs, with the exception of Na2S2O3. Additionally, it could fix CO_2_ using the incomplete CBB cycle. However, it did not have the capacity to oxidize iron and fix nitrogen. We also constructed the first high-quality metabolic model for *Acidithiobacillus Ameehan*. The model is comprised of 744 genes, 1,374 metabolites, and 1,377 reactions that are distributed among the extracellular, periplasmic, and cytoplasmic compartments, which allowed us to investigate the unique sulfur oxidation pathways and the interconnected carbon fixation pathways of the strain. Through rigorous validation against experimental data and existing literature, we observed a high degree of agreement between the model and experimental results (88.7% agreement with experimental Biolog data). The model showed five potential sulfur oxidation pathways for *Acidithiobacillus Ameehan* using sulfur as a substrate. The optimal sulfur oxidation pathway showed the highest ATP production rate of 14.81 mmol/gDW/h and NADH/NADPH production rate of 5.76 mmol/gDW/h, with CO_2_ consumption of 1.575 mmol/gDW/h and sulfur consumption of 1.5 mmol/gDW/h. By analyzing the commonalities among these sulfur oxidation pathways, we found two crucial genes, OMP and SQR, which played critical roles in cellular sulfur oxidation and significantly enhanced sulfur oxidation efficiency. These findings provided valuable insights into the growth and metabolism of various substances by *Acidithiobacillus Ameehan*. Moreover, the constructed model serves as a convenient platform for future engineering efforts aimed at modifying the strain.

## Data availability statement

The original contributions presented in the study are included in the article/Supplementary material. The data presented have been deposited in GitHub: https://github.com/wupeng1998/Acidithiobacillus-Ameehan.

## Author contributions

PW: Data curation, Formal Analysis, Methodology, Writing – original draft. QY: Formal Analysis, Methodology, Writing – original draft, Funding acquisition, Writing – review & editing. TC: Formal Analysis, Writing – original draft, Data curation. YH: Formal Analysis, Investigation, Methodology, Writing – original draft. WZ: Data curation, Formal Analysis, Investigation, Writing – original draft. XL: Formal Analysis, Writing – original draft, Methodology. LW: Formal Analysis, Writing – original draft, Data curation, Investigation. JC: Formal Analysis, Investigation, Writing – original draft, Funding acquisition. QH: Investigation, Writing – original draft, Data curation, Methodology. YG: Data curation, Investigation, Writing – original draft, Formal Analysis. XZ: Data curation, Formal Analysis, Writing – original draft. FL: Project administration, Resources, Writing – review & editing. JW: Project administration, Writing – review & editing, Funding acquisition, Methodology. HM: Funding acquisition, Project administration, Writing – review & editing. ZH: Funding acquisition, Project administration, Writing – review & editing.
