# Supplementary material for: Genome sequencing and metabolic network reconstruction of a novel sulfur-oxidizing bacterium Acidithiobacillus Ameehan
Source: Front Microbiol. 2023 Nov 20;14:1277847. doi: 10.3389/fmicb.2023.1277847 (PMC10694236; doi:10.3389/fmicb.2023.1277847)
Supplement: Supplementary file 10 [file Presentation_1.pdf]

A genome metabolic network model of new sulfur-oxidizing bacterium *Acidithiobacillus* sp. AMEEHan

Peng Wu[1,2,4†], Qianqian Yuan[3,4†], Yifan Han[2,4†], Wei Zhao[2,4†], Tingting Cheng[1,2,4], Xiaoping Liao[3,4], Lu Wang[1,2,4], Jingyi Cai[3,4], Qianqian He, Ying Guo[2,4], Xiaoxia Zhang[2,4], Fuping Lu[1], Jingjing Wang[2,4*], Hongwu Ma[3,4*], Zhiyong Huang[2,4*]

[1]College of Bioengineering, Tianjin University of Science and Technology, Tianjin, China [2]Tianjin Key Laboratory for Industrial Biological Systems and Bioprocessing Engineering, Tianjin Institute of Industrial Biotechnology, Chinese Academy of Sciences, Tianjin, China
[3]National Technology Innovation Center of Synthetic Biology, Tianjin, China
[4]Core Facility, Tianjin Institute of Industrial Biotechnology, Chinese Academy of Sciences, Tianjin, China

† These authors contributed equally to this work.

*** Correspondence:**
Hongwu Ma
ma_hw@tib.cas.cn

Jingjing Wang
wang_jj@tib.cas.cn

Zhiyong huang
huang_zy@tib.cas.cn

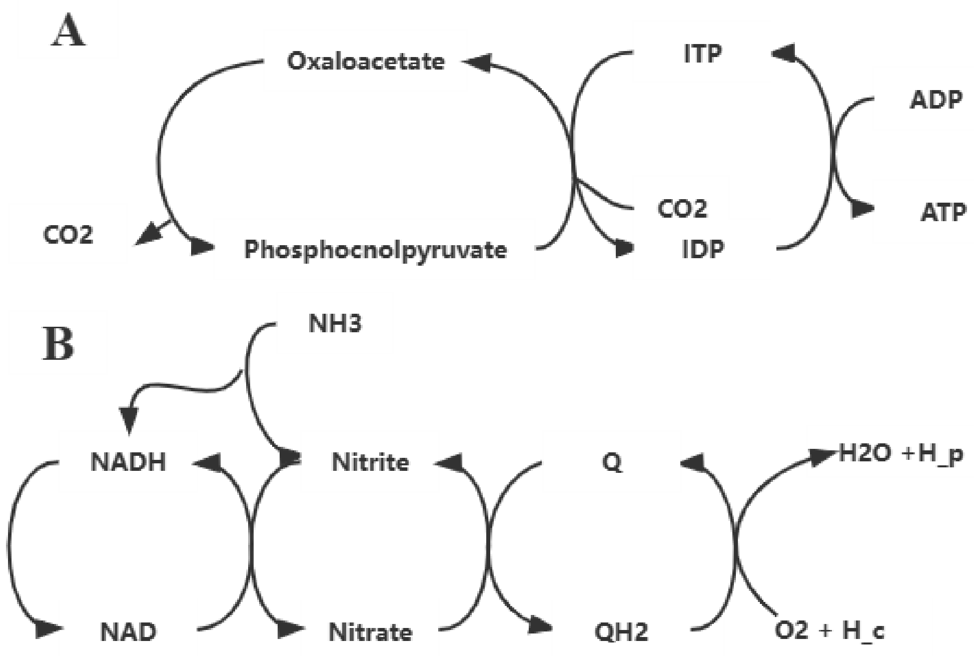

Supplementary Figure S1. Two type errors in Model_01. (A) The energy-generation cycle leading to ATP inifite generation. (B) The proton pump cycle leading to ATP infinite generation.

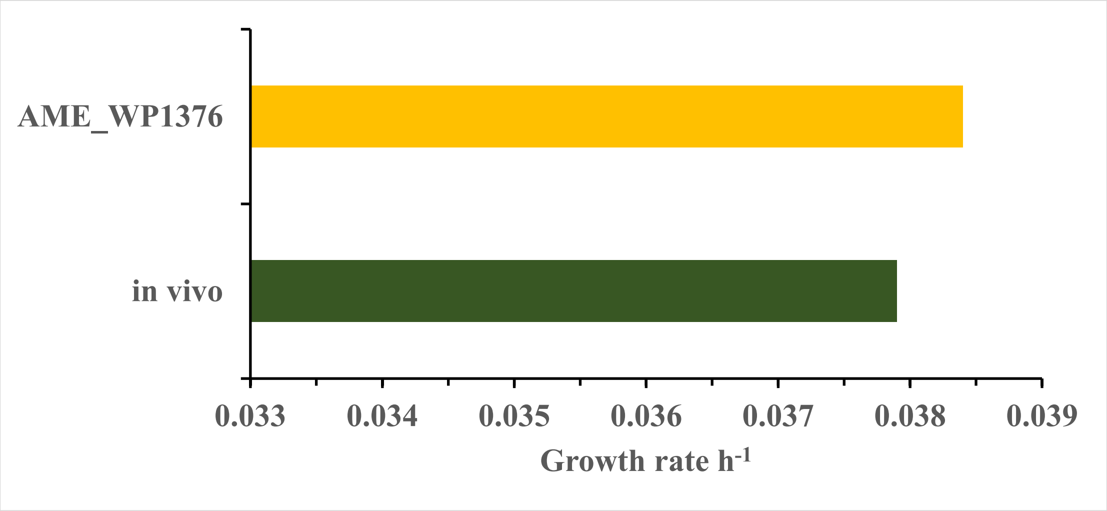

Supplementary Figure S2. Comparison of *in vivo* and *in silico* growth rates of *Acidithiobacillus* sp. AMEEHan.

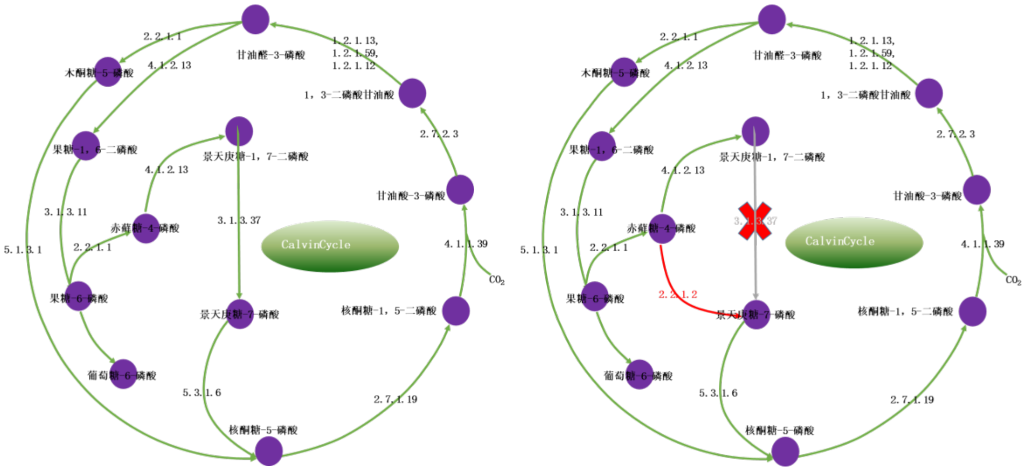

Supplementary Figure S3. Differences between real Calvin and variant Calvin cycles.

The left diagram shows the actual Calvin cycle carbon sequestration metabolic pathway, and the right diagram shows the Calvin carbon sequestration metabolic pathway of AC bacteria variant, its Calvin cycle lacks the ability to catalyze the reaction of Sedum heptonulose 1,7-diphosphate into Sedum heptonulose-7-phosphate during the regeneration of RuBP in the third step of the Calvin cycle, but it can converting erythrose 4-phosphate into Sedum heptonulose-7-phosphate, which enables the Calvin cycle to proceed.
